# Liposomal Delivery Enhances the Effects of a Collagen Tripeptide–Containing Formulation on Dermal Structure and Optical Skin Parameters: A Randomized, Double‐Blind, Placebo‐Controlled Trial

**DOI:** 10.1111/jocd.70834

**Published:** 2026-03-31

**Authors:** Yung‐Kai Lin, Chia‐Hua Liang, Ban‐Chin Huang, Sze‐Huey Lim, Yung‐Hsiang Lin, Shu‐Ting Chan, Chi‐Fu Chiang, Yi‐Wen Mao

**Affiliations:** ^1^ Institute of Food Safety and Risk Management, National Taiwan Ocean University Keelung Taiwan; ^2^ Department of Food Science National Taiwan Ocean University Keelung Taiwan; ^3^ Graduate Institute of Biomedical Engineering, National Chung Hsing University Taichung Taiwan; ^4^ Department of Cosmetic Science and Institute of Cosmetic Science Chia Nan University of Pharmacy and Science Tainan Taiwan; ^5^ BW Research & Innovation Unit Singapore; ^6^ Research & Design Center, TCI Co. Ltd. Taipei Taiwan

**Keywords:** collagen tripeptides, dermal collagen, liposomal delivery, skin elasticity, skin luminance, skin physiology

## Abstract

**Objective:**

This study aimed to determine whether liposomal delivery enhances the effects of a collagen tripeptide‐containing formulation on dermal structural and biomechanical parameters, as well as appearance‐related skin properties, compared with a nonliposomal formulation and placebo.

**Methods:**

In a randomized, double‐blind, placebo‐controlled trial, 75 healthy adults aged 25–65 years were assigned to receive placebo, a nonliposomal formulation containing collagen tripeptides, or a liposomal formulation containing collagen tripeptides (50 mL/day) for 8 weeks. Objective assessments of dermal collagen density, skin hydration, elasticity, wrinkle area, skin luminance, and tone evenness were performed at baseline and Weeks 2, 4, and 8 using standardized instrumental measurements.

**Results:**

Both collagen tripeptide‐containing formulation groups showed significant improvements in dermal collagen density, hydration, and elasticity compared with placebo (*p* < 0.05). The liposomal formulation demonstrated an earlier onset and a greater magnitude of improvement across multiple parameters, with a statistically significant advantage in skin elasticity at Week 8 compared with the nonliposomal formulation (*p* < 0.05). Notably, only the liposomal collagen tripeptide‐containing formulation group exhibited a significant reduction in wrinkle area at Week 8 relative to placebo (*p* < 0.05). Both collagen tripeptide formulation groups also showed significant increases in skin luminance and tone evenness.

**Conclusion:**

Liposomal delivery significantly enhances the functional effects of a collagen tripeptide‐containing formulation on dermal structure and biomechanical performance, particularly dermal collagen density and skin elasticity, as well as optical skin properties including luminance and tone evenness. These findings support the application of liposomal formulations containing collagen tripeptides as an effective oral nutricosmetic strategy for improving visible signs of skin aging.

**Trial Registration:**

ClinicalTrials.gov identifier: NCT06771388

## 1 Introduction

Skin aging is a multifactorial biological process influenced by both intrinsic and extrinsic factors, including chronological aging, ultraviolet (UV) exposure, oxidative stress, hormonal alterations, and nutritional status. These factors collectively disrupt dermal extracellular matrix (ECM) homeostasis, leading to reduced collagen synthesis, impaired elasticity, decreased hydration, and the progressive development of wrinkles and uneven skin appearance [1]. Among ECM components, collagen plays a central role in maintaining dermal structural integrity, mechanical strength, and tissue resilience, and age‐related declines in collagen production contribute to the visible and functional manifestations of skin aging [2].

Oral supplementation with collagen‐derived peptides has been investigated as a strategy to support dermal structure from within. Experimental and clinical studies have shown that low‐molecular‐weight collagen peptides, including collagen tripeptides, can be absorbed through the intestinal epithelium and modulate dermal fibroblast activity, extracellular matrix synthesis, and skin barrier‐related functions under physiological and stress conditions [3]. Nevertheless, the functional efficacy of orally administered peptides may in part be constrained by enzymatic degradation and limited bioavailability within the gastrointestinal tract, highlighting the importance of delivery strategies that enhance peptide stability and absorption [4].

Liposomal delivery systems have emerged as promising approaches to improve the stability, permeability, and bioavailability of bioactive compounds. Liposomes composed of phospholipid bilayers can encapsulate peptides, protect them from enzymatic hydrolysis, and facilitate controlled release and cellular uptake, as demonstrated across pharmaceutical, nutritional, and food science applications [5, 6]. Although liposomal formulations have been extensively explored in experimental and topical contexts, clinical evidence directly comparing liposomal and nonliposomal oral formulations containing collagen tripeptides with respect to skin functional outcomes remains limited [7, 8].

In addition to collagen peptides, skin function is influenced by multiple bioactive components involved in hydration, antioxidative defense, and cellular metabolism [9]. Yeast extract contains nucleotides, amino acids, and β‐glucans that have been reported to support cellular metabolism, immune modulation, and barrier‐related functions in diverse biological systems [10]. Hyaluronic acid (HA), a major dermal glycosaminoglycan, contributes to tissue hydration and viscoelasticity, and oral HA supplementation has been associated with improvements in skin hydration, elasticity, and wrinkle‐related parameters in human studies [11]. Resveratrol, a polyphenolic compound, exhibits antioxidant and cytoprotective properties that mitigate oxidative stress and inflammatory responses in multiple experimental models, suggesting potential relevance to skin aging processes [12].

Together, the combination of these bioactive components with collagen peptides is expected to support dermal function through complementary and potentially synergistic biological pathways, serving as a supportive formulation background rather than independent intervention factors in the present study.

Therefore, the present randomized, double‐blind, placebo‐controlled clinical study aimed to evaluate whether liposomal delivery enhances the effects of a formulation containing collagen tripeptides on dermal matrix‐related, biomechanical, and clinically relevant skin appearance parameters, compared with a nonliposomal formulation, in healthy adults.

## 2 Material and Methods

### 2.1 Preparation of Test Formulations

The test formulations used in this study were beverages containing fish‐derived collagen tripeptides. The collagen tripeptides were produced by enzymatic hydrolysis of fish collagen and consisted predominantly of Gly–X–Y tripeptide sequences characteristic of collagen‐derived peptides.

Two types of formulations were prepared: a nonliposomal formulation and a liposomal formulation containing collagen tripeptides. In the nonliposomal formulation, collagen tripeptides were dissolved directly in the beverage matrix without encapsulation.

For the liposomal formulation, collagen tripeptides were incorporated into phospholipid vesicles using a high‐pressure homogenization process. The homogenization was conducted at 400 bar to promote uniform vesicle formation, improve encapsulation stability, and enhance the structural integrity of the liposomal system.

### 2.2 Clinical Trial Design

**FIGURE 1:**
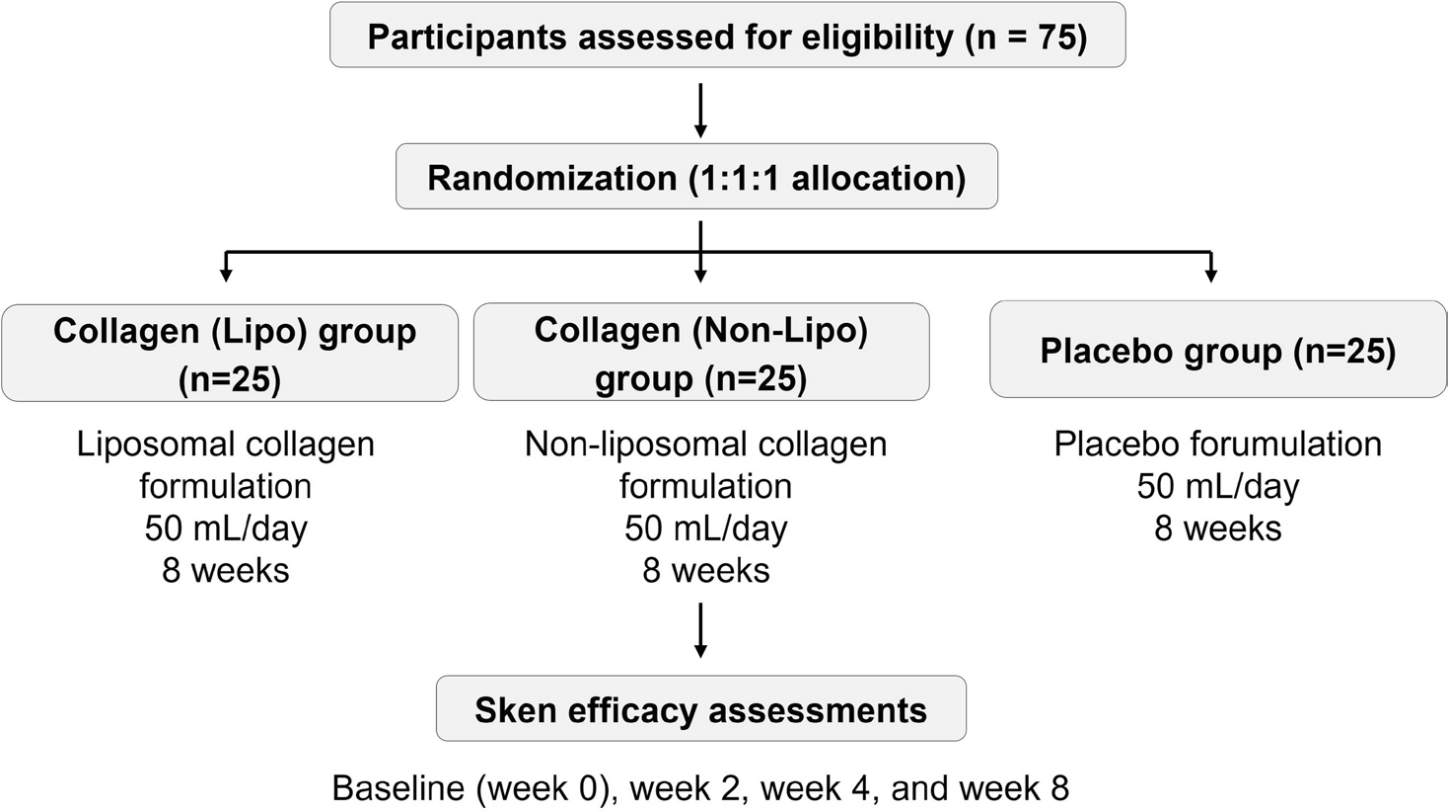
Schematic diagram of the study design and participant grouping.

The study was registered in ClinicalTrials.gov (No. NCT06771388), was conducted under a protocol approved by an independent Institutional Review Board, and was performed in accordance with the Declaration of Helsinki (1964) and its amendments. Written informed consent was obtained from all participants after a full explanation of the study. A double‐blind, placebo‐controlled, randomized study was conducted. A total of 75 healthy male and female subjects aged 25–65 years were recruited for this study. Eligible subjects were those aged 25–65 years who voluntarily agreed to participate after providing written informed consent. Subjects were excluded if they met any of the following conditions: nonvoluntary participation or unwillingness to comply with the study procedures; clinically diagnosed dermatological diseases, liver cirrhosis, or chronic renal failure; known allergies to cosmetic products, drugs, or food ingredients used in the study formulations; pregnancy or lactation; current use of medications for chronic diseases; having received facial treatments such as laser therapy, chemical or fruit acid peeling, or excessive sun exposure (more than 3 h per day within the past week) within 8 weeks prior to enrollment; or being students directly taught or academically supervised by the principal investigator. Subjects were randomly assigned into three groups (*n* = 25 per group) using a computer‐generated randomization sequence: 1. Placebo group: received a placebo beverage of the same appearance and flavor without active ingredients; 2. Collagen (non‐Lipo) group: received a nonliposomal formulation containing collagen tripeptides of identical composition except for the liposomal modification; 3. Collagen (Lipo) group: received a liposomal formulation containing collagen tripeptides. Subjects were instructed to consume 50 mL of their assigned test sample once daily before bedtime for eight consecutive weeks. Objective assessments of skin condition and subjective evaluations were conducted at baseline (week 0), Week 2, Week 4, and Week 8. Subjective evaluations were performed simultaneously using a self‐administered questionnaire that assessed subjects' perceived improvements in skin condition. The study design and participant grouping are illustrated in Figure 1.

### 2.3 Supplement Formulation

Three beverage formulations were prepared for the intervention: a liposomal formulation containing collagen tripeptides (Collagen Lipo), a nonliposomal formulation containing collagen tripeptides (Collagen Non‐Lipo), and a placebo formulation.

Collagen (Lipo): A liposomal formulation containing fish‐derived collagen tripeptides incorporated into a phospholipid‐based vesicle delivery system (Lipo‐MAXI collagen) designed to encapsulate collagen tripeptides and enhance their stability and bioavailability, yeast extract powder, sodium hyaluronate, resveratrol, apple juice, citric acid, malic acid, steviol glycosides, water, flavor, erythritol, and sucralose. This formulation corresponds to a commercially available oral collagen supplement (Avance QQ Collagen).

Collagen (Non‐Lipo): A nonliposomal formulation containing fish‐derived collagen tripeptides (MAXI collagen), yeast extract powder, sodium hyaluronate, resveratrol, apple juice, citric acid, malic acid, steviol glycosides, water, flavor, erythritol, and sucralose.

Placebo: Apple juice, citric acid, malic acid, steviol glycosides, water, flavor, erythritol, and sucralose.

The liposomal collagen formulation, nonliposomal collagen formulation, and placebo beverages were identical in appearance, flavor, and packaging, with the same bottle shape, size, and color and were coded by an independent staff member not involved in outcome assessment, to ensure blinding of both subjects and investigators.

### 2.4 Clinical Skin Efficacy Assessment

Objective skin measurements were performed using standardized dermatological instruments under controlled temperature (22°C ± 2°C) and humidity (50% ± 10%) conditions following a 20‐min acclimation period, with all measurements conducted on the left cheek unless otherwise specified.

#### 2.4.1 Skin Luminance and Tone Uniformity

Skin luminance and tone uniformity were evaluated using a chromameter (Chroma Meter MM500, Minolta, Japan); the individual typology angle (ITA°) and L* values were calculated according to the CIE Lab* color system to represent overall skin radiance and evenness, where higher L* and ITA° values indicate lighter and brighter skin tones.

#### 2.4.2 Skin Hydration

Skin hydration was measured using a corneometer (Corneometer CM825, CK, Germany) based on electrical capacitance to assess the water‐retention capacity of the stratum corneum.

#### 2.4.3 Skin Elasticity

Skin elasticity was assessed using a cutometer (Cutometer MPA580, CK, Germany), which measures skin deformation under negative pressure and evaluates the viscoelastic recovery parameters R5 (net elasticity).

#### 2.4.4 Wrinkle Analysis

Facial and periorbital wrinkles were analyzed using a digital facial imaging analyzer (VISIA Complexion Analysis, Canfield Scientific, USA) with standardized lighting and 360‐degree high‐resolution photography to quantify wrinkle distribution and depth, where darker areas represent regions of increased wrinkle depth and density.

#### 2.4.5 Skin Collagen Density

Skin collagen density was determined using an ultrasound skin analyzer (DermaLab Series SkinLab Combo, Cortex, Denmark); the collagen index was quantified by measuring dermal echogenicity and reflection intensity on the cheek area.

#### 2.4.6 Subjective Evaluation

Subjects also completed self‐administered questionnaires to evaluate perceived improvements in skin condition.

All measurements were performed by trained technicians using standardized procedures to minimize interoperator variability.

### 2.5 Statistical Analysis

The comparison of measurement results for skin parameters within groups at each time point was analyzed using paired *t*‐tests, and comparisons among groups at corresponding time points were performed using one‐way analysis of variance (ANOVA). All statistical analyses were conducted using GraphPad Prism, and *p* < 0.05 was considered statistically significant.

## 3 Results

### 3.1 Liposomal Collagen Tripeptide Increased Skin Collagen, Hydration, and Elasticity

Baseline demographic characteristics are summarized in Table 1. Each group (placebo, collagen (non‐Lipo), and collagen (Lipo)) included 25 subjects. The mean ages were 46.6 ± 17.3, 47.6 ± 14.8, and 46.0 ± 16.7 years, respectively, and sex distribution was comparable among groups (female/male: 22/3, 24/1, and 20/5).

**TABLE 1:** Baseline characteristics of subjects.

Parameter	Placebo group	Collagen (non‐Lipo) group	Collagen (Lipo) group
Number of subjects (*n*)	25	25	25
Sex (F/M)	22/3	24/1	20/5
Age (years)			
Mean ± SD	46.6 ± 17.3	47.6 ± 14.8	46.0 ± 16.7

**FIGURE 2:**
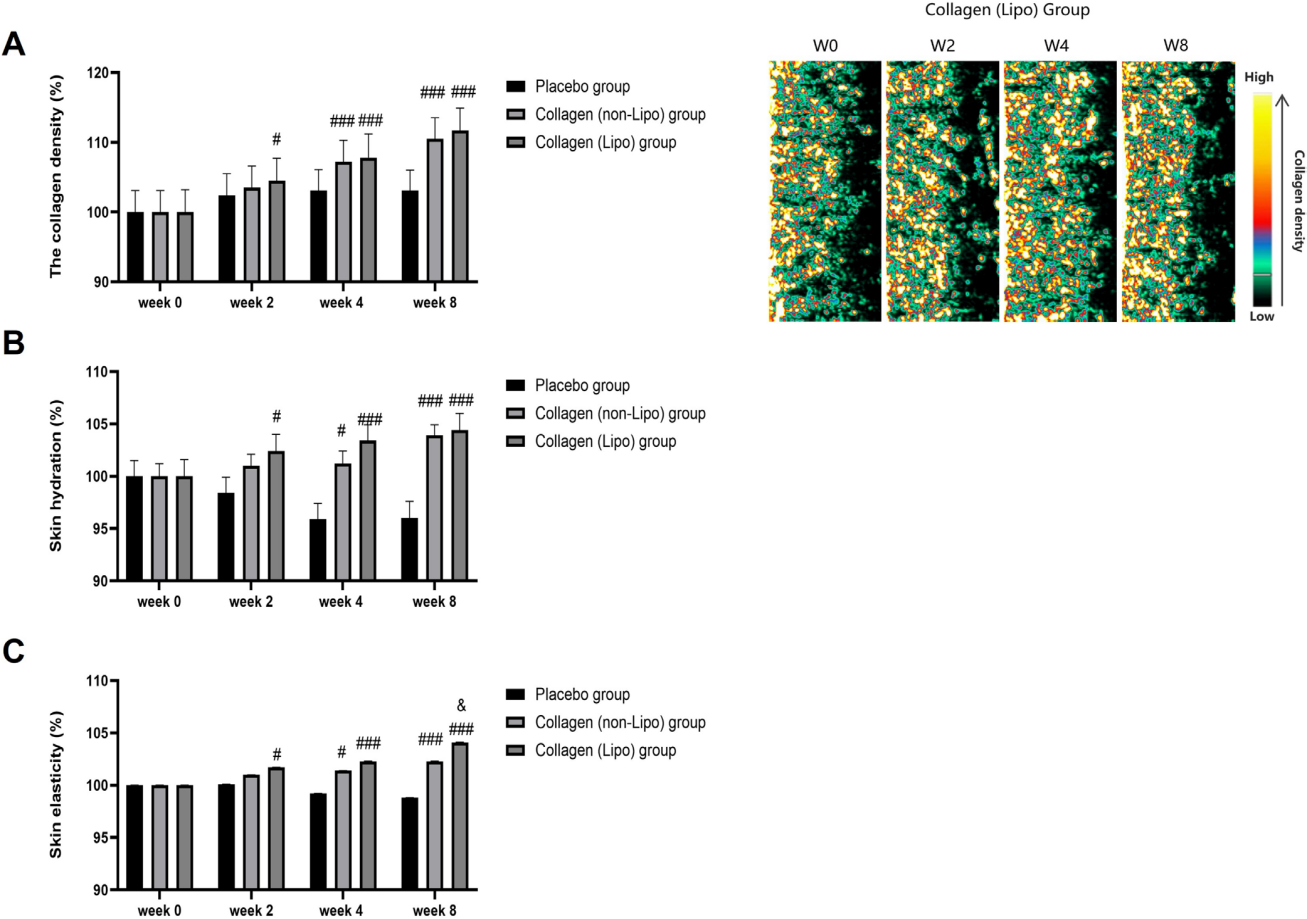
Effects of oral liposomal collagen tripeptides on skin collagen density, hydration, and elasticity. (A) Skin collagen density (%). Representative echogenicity images of the collagen (Lipo) group are shown on the right, with yellow‐to‐red regions indicating high collagen density and green‐to‐blue regions indicating low density. (B) Skin hydration (%). (C) Skin elasticity (%). Data are presented as mean ± SEM. ^#^ *p* < 0.05, ^##^ *p* < 0.01, ^###^ *p* < 0.001 vs. placebo group; ^&^ *p* < 0.05 vs. collagen (non‐Lipo) group.

In terms of skin collagen density (Figure 2A), the collagen (Lipo) group showed a significant increase compared with the placebo group from week 2 (^#^ *p* < 0.05). At Weeks 4 and 8, both the collagen (Lipo) and collagen (non‐Lipo) groups exhibited significantly higher collagen density than the placebo group (^###^ *p* < 0.001). Across Weeks 2, 4, and 8, the collagen (Lipo) group consistently demonstrated slightly higher mean collagen density values than the collagen (non‐Lipo) group. Representative ultrasound images further illustrated increased dermal echogenicity after 8 weeks in the collagen (Lipo) group, reflected by a color shift from low‐density (green) to higher‐density (yellow to red) regions.

Regarding skin hydration (Figure 2B), the collagen (Lipo) group showed a significant improvement as early as Week 2 compared with the placebo group (^#^ *p* < 0.05), with further increases observed at Weeks 4 and 8 (^###^ *p* < 0.001). The collagen (non‐Lipo) group also exhibited significant improvements at Weeks 4 and 8 (^###^ *p* < 0.001) relative to the placebo group. Across Weeks 2, 4, and 8, mean skin hydration levels in the collagen (Lipo) group were consistently slightly higher than those in the collagen (non‐Lipo) group, indicating a trend toward enhanced stratum corneum water retention associated with the liposomal formulation.

Skin elasticity results are shown in Figure 2C. The collagen (Lipo) group demonstrated a significant increase in skin elasticity at Week 2 compared with the placebo group (^#^ *p* < 0.05), with further improvements at Weeks 4 and 8 (^###^ *p* < 0.001). By Week 8, the increase in skin elasticity in the collagen (Lipo) group was significantly greater than that observed in the collagen (non‐Lipo) group (^&^ *p* < 0.05), indicating that supplementation with the liposomal formulation containing collagen tripeptides resulted in a more pronounced improvement in skin biomechanical properties.

Collectively, these results indicate that oral supplementation with formulations containing collagen tripeptides improved dermal collagen density, hydration, and elasticity, with liposomal delivery associated with an earlier onset and a consistently greater magnitude of improvement across these parameters.

### 3.2 Liposomal Collagen Tripeptide Formulation Decreased Skin Wrinkles and Increased Whitening, Tone‐Lightening

**FIGURE 3:**
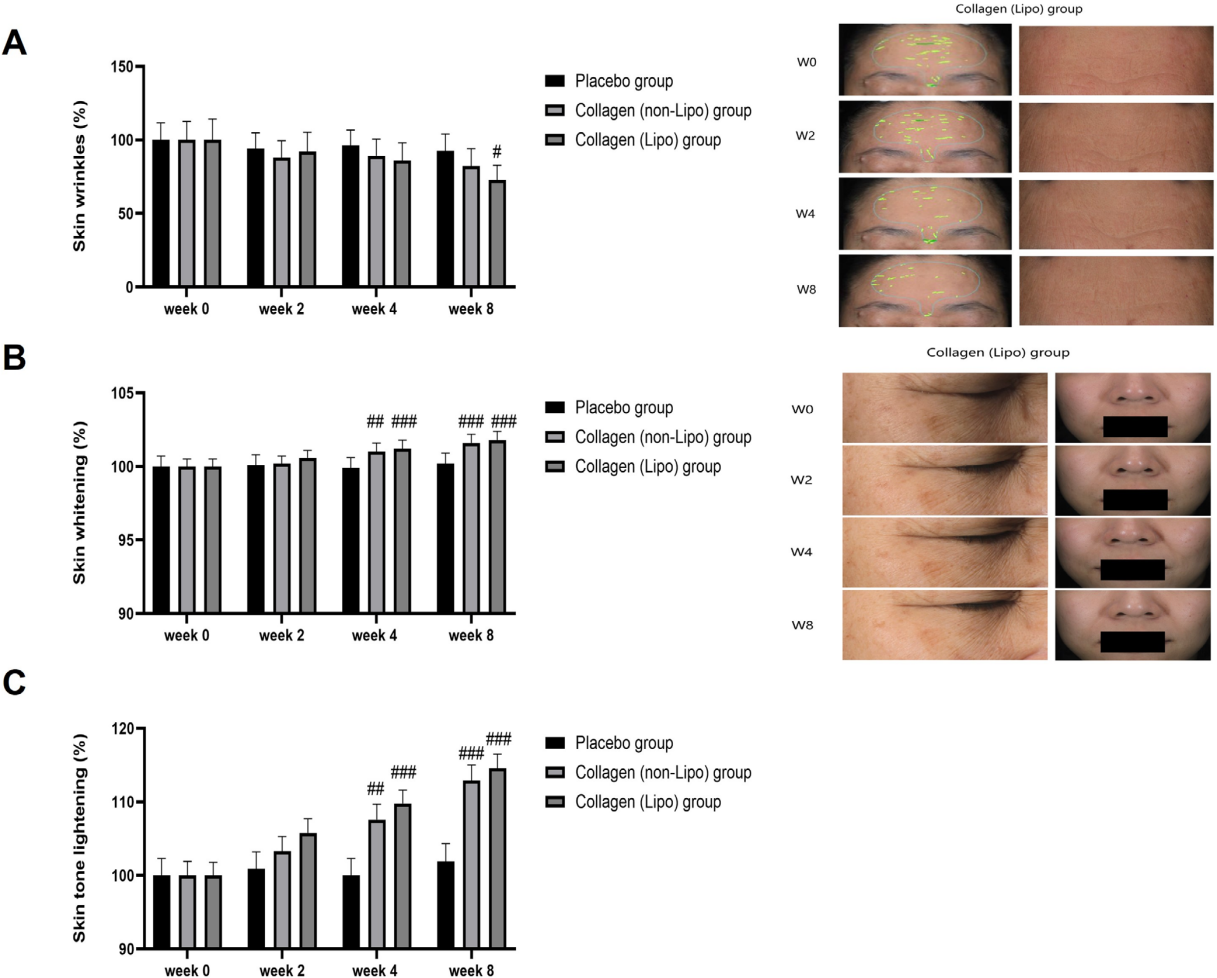
Effects of oral liposomal collagen tripeptides on skin wrinkles, whitening, and tone lightening. (A) Skin wrinkle area (%). Representative images of the collagen (Lipo) group show gradual reductions in wrinkle depth and density from baseline (W0) to Week 8 (W8). (B) Skin whitening (%), with representative facial photographs illustrating improvements in overall brightness in the collagen (Lipo) group. (C) Skin tone lightening (%). Data are presented as mean ± SEM. ^#^ *p* < 0.05, ^##^ *p* < 0.01, ^###^ *p* < 0.001 vs. placebo group.

In terms of skin wrinkles (Figure 3A), the collagen (Lipo) group exhibited a significant reduction in wrinkle area at Week 8 compared with the placebo group (^#^ *p* < 0.05), whereas no statistically significant changes were observed in the collagen (non‐Lipo) or placebo groups throughout the intervention period. Representative facial images further demonstrated visible improvements in wrinkle depth and density in the periorbital region after 8 weeks of supplementation in the collagen (Lipo) group, consistent with a smoother skin surface appearance.

Regarding skin luminance (Figure 3B), both the collagen (Lipo) and collagen (non‐Lipo) groups showed significantly higher luminance values compared with the placebo group at Weeks 4 and 8 (^#^ *p* < 0.05; ^###^ *p* < 0.001). At Week 4, the increase in skin luminance was numerically more pronounced in the collagen (Lipo) group relative to the collagen (non‐Lipo) group. Across Weeks 2, 4, and 8, the mean luminance values in the collagen (Lipo) group were consistently numerically higher than those in the collagen (non‐Lipo) group, indicating a trend toward enhanced skin brightness with liposomal delivery. Representative photographs also illustrated visibly brighter and more uniform facial skin tone in subjects receiving the liposomal formulation.

Skin tone lightening outcomes are presented in Figure 3C. The collagen (Lipo) group demonstrated a progressive increase in skin tone lightening from Week 2, with statistically significant differences compared with the placebo group observed at Weeks 4 and 8 (^###^ *p* < 0.001). The collagen (non‐Lipo) group exhibited a similar pattern, showing significant improvements relative to the placebo group at Weeks 4 and 8 (^##^ *p* < 0.01; ^###^ *p* < 0.001). Across all assessment time points, the mean skin tone lightening values in the collagen (Lipo) group remained slightly higher than those in the collagen (non‐Lipo) group, suggesting a consistent numerical advantage associated with liposomal delivery.

Collectively, these findings indicate that oral supplementation with formulations containing collagen tripeptides was associated with reductions in wrinkle area and improvements in skin luminance and tone lightening, with liposomal delivery showing a consistent numerical advantage and more pronounced effects for selected outcome parameters, particularly wrinkle area reduction at Week 8.

### 3.3 Subjective Evaluation of Skin Condition Based on Self‐Assessment Questionnaire

**FIGURE 4:**
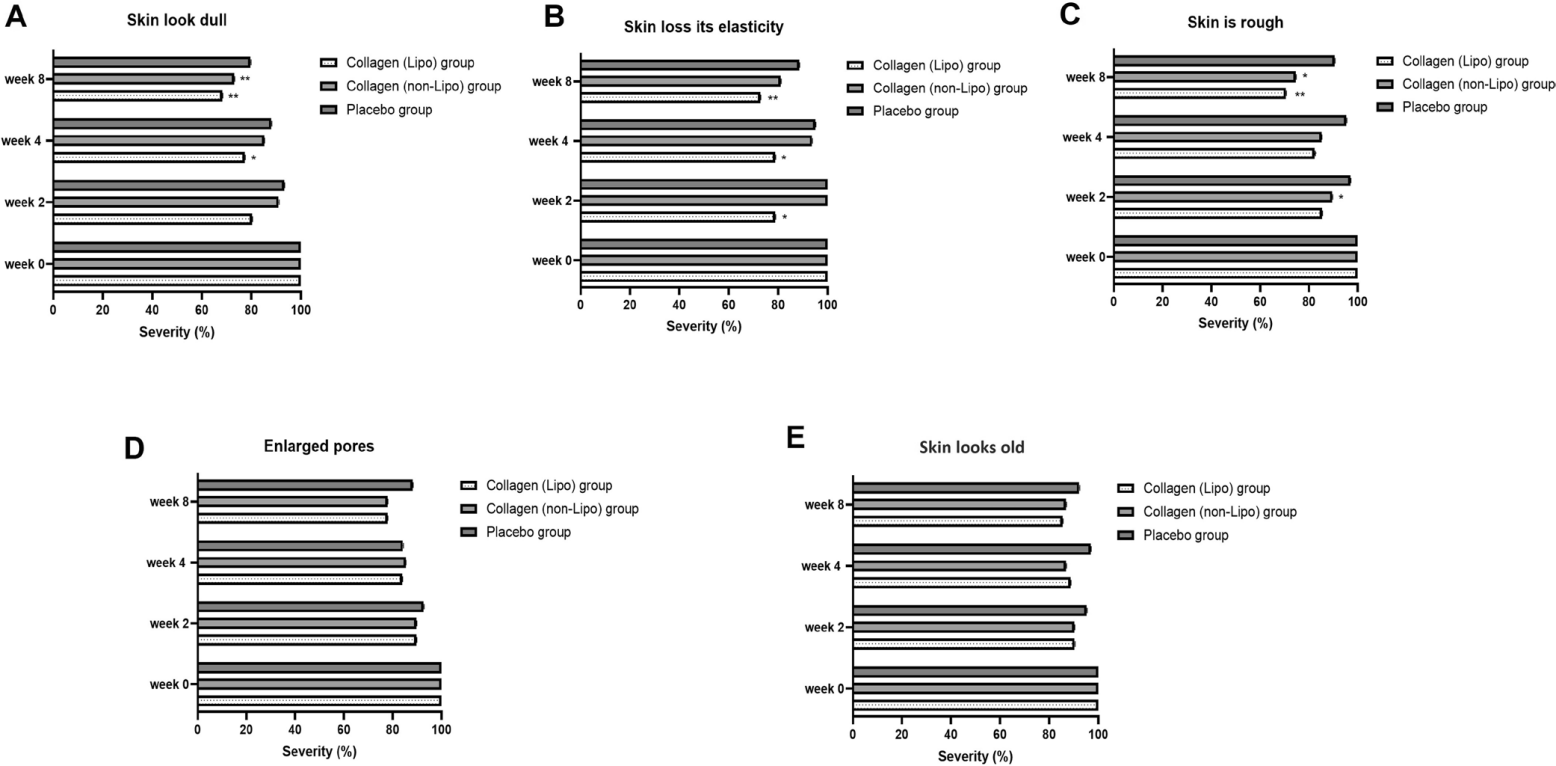
Subjective evaluation of skin condition after 8 weeks of oral supplementation with liposomal collagen tripeptides. Subjects completed a self‐assessment questionnaire at baseline (Week 0), Week 2, Week 4, and Week 8 to evaluate perceived changes in skin condition. Ratings of severity (%) were collected for (A) skin dullness, (B) loss of elasticity, (C) roughness, (D) enlarged pores, and (E) skin looking old. Data are presented as mean ± SEM. *p < 0.05, **p < 0.01 vs. baseline.

Subjective perceptions of skin condition were assessed using a self‐administered questionnaire, and the results are presented in Figure 4.

For perceived skin dullness (Figure 4A), a significant reduction in dullness severity was observed in the collagen (Lipo) group at Week 4 (*p < 0.05 vs. baseline) and became more pronounced at Week 8 (**p < 0.01). At Week 8, the collagen (non‐Lipo) group also exhibited a significant reduction compared with baseline (**p < 0.01). Across the assessed time points, the collagen (Lipo) group consistently showed lower mean dullness scores than the collagen (non‐Lipo) group, suggesting a trend toward greater perceived improvement in skin brightness.

Regarding perceived loss of skin elasticity (Figure 4B), the collagen (Lipo) group demonstrated a significant decrease in severity at Week 2 (*p < 0.05 vs. baseline), with sustained improvements at Week 4 (*p < 0.05) and Week 8 (**p < 0.01). No significant changes from baseline were observed in the collagen (non‐Lipo) group during the intervention period. These findings indicate an earlier and more consistent perceived improvement in skin elasticity in subjects receiving the liposomal collagen tripeptide formulation.

For perceived skin roughness (Figure 4C), a significant reduction from baseline was observed in the collagen (non‐Lipo) group at Week 2 (*p < 0.05). At Week 8, both the collagen (Lipo) (**p < 0.01) and collagen (non‐Lipo) (*p < 0.05) groups exhibited significant decreases in roughness severity compared with baseline. The degree of improvement was greater in the collagen (Lipo) group, indicating that subjects perceived a smoother and finer skin texture following liposomal collagen tripeptide formulation supplementation.

In contrast, no significant changes were observed for perceived enlarged pores (Figure 4D) or skin looking old (Figure 4E) in any group during the 8‐week intervention, indicating minimal perceived differences among subjects in these aspects.

## 4 Discussion

This clinical study demonstrated that oral supplementation with the liposomal collagen tripeptide formulation was associated with favorable improvements in multiple skin parameters related to aging and appearance. After 8 weeks of intake, both the collagen (non‐Lipo) and collagen (Lipo) groups showed significant improvements in skin collagen density, hydration, and elasticity compared with the placebo. When the two active formulations were compared, the liposomal formulation consistently showed a greater magnitude of improvement across several parameters, and a statistically significant between‐formulation difference was observed for skin elasticity at Week 8. The liposomal collagen tripeptide formulation group also exhibited a significant reduction in wrinkle area, whereas no statistically significant reduction in wrinkle area was observed in the nonliposomal formulation group during the intervention period. Similarly, for skin collagen density, hydration, luminance, and tone lightening, the liposomal formulation generally showed numerically greater improvements than the non‐liposomal formulation, although these between‐formulation differences did not reach statistical significance. Furthermore, subjective evaluations were consistent with instrumental findings, as subjects reported decreased skin dullness, improved elasticity, and smoother texture. Overall, these results suggest that supplementation with the liposomal collagen tripeptide formulation was associated with measurable and perceivable improvements in skin parameters within an 8‐week period, with the most evident advantage over the nonliposomal formulation observed for skin elasticity and wrinkle‐related outcomes.

The improvements observed in dermal collagen density, skin elasticity, and wrinkle appearance may be biologically interconnected. Increased collagen deposition within the dermal extracellular matrix enhances the structural integrity and mechanical resilience of the skin, which in turn contributes to improved elasticity and reduced wrinkle formation. Strengthening of the dermal matrix is widely recognized as a key mechanism underlying visible improvements in skin smoothness and texture. These relationships may provide a plausible biological explanation for the concurrent improvements in collagen density, elasticity, and wrinkle‐related parameters observed in the present study.

The collagen tripeptides (non‐Lipo) formulation contained several bioactive ingredients—collagen tripeptide, yeast extract powder, sodium hyaluronate, and resveratrol—that may contribute to improvements in skin structure, hydration, and barrier function [13]. Collagen tripeptides have been reported to stimulate dermal fibroblast activity, promote the synthesis of collagen and elastin, and enhance skin firmness and hydration [14]. Due to their low molecular weight, collagen tripeptides may be efficiently absorbed through the intestinal barrier and promote the expression of genes such as COL1A1 and ELN, thereby supporting extracellular matrix stability and integrity [14, 15]. In addition, collagen tripeptides may increase endogenous hyaluronic acid production in the skin, contributing to improved moisture retention and barrier integrity [16].

Yeast extract contains peptides, β‐glucans, and amino acids that may support keratinocyte metabolic activity and skin barrier function [10]. Sodium hyaluronate is known to contribute to skin hydration and biomechanical properties. Clinical studies have demonstrated that sodium hyaluronate supplementation may improve skin hydration and smoothness [17]. Resveratrol is a polyphenolic antioxidant that may help protect the skin against oxidative stress and has been associated with protective effects against oxidative stress–related skin aging [18, 19].

Previous studies showed that liposomal delivery enhanced the efficacy of collagen peptides on skin health through multiple complementary mechanisms [13]. The lipid bilayer provided structural stability and enzymatic protection in aqueous and acidic environments, reducing gastrointestinal degradation and preserving the integrity of functional peptides [4]. The surface charge and nanoscale size of liposomes (typically within the 100 nm range reported in most studies) facilitated lipophilic interactions and endocytosis with intestinal epithelial membranes, thereby increasing local contact and residence time between peptides and epithelial cells [20]. In addition, liposomes were engineered to provide sustained or layered‐release kinetics, allowing active peptides to maintain effective concentrations within the intestinal tract for extended periods and to exert prolonged physiological effects through skin‐related pathways—such as stimulating ECM remodeling signals in dermal fibroblasts and inhibiting oxidative stress–induced matrix degradation [21]. Recent studies further supported these mechanistic insights. Liposomal encapsulation improved peptide stability, mucosal permeation, and intestinal retention, thereby potentially enhancing functional bioavailability [22]. Although most investigations had focused on pharmacological peptides, the same principles may be applicable to nutraceutical bioactive peptides such as collagen tripeptides [23]. Complementary in vitro and ex vivo studies demonstrated that liposome‐encapsulated collagen or tripeptides showed higher deposition within skin layers, increased retention of structural proteins including collagen, and greater activation of dermal fibroblast function compared with nonencapsulated forms [7]. Furthermore, liposomal systems incorporating antioxidant or anti‐aging peptides, such as GHK‐Cu, exhibited enhanced transdermal permeability and bioactivity relative to conventional formulations [8]. Collectively, these findings suggested that the liposomal delivery system conferred multiple advantages—including protection from degradation, controlled release, epithelial interaction, and enhanced tissue accessibility—which together provided a biologically plausible explanation for the earlier onset and greater magnitude of improvement observed in skin collagen density, hydration, and elasticity in the liposomal collagen tripeptide formulation group of the present study.

A key design feature of this study was that the non‐Lipo and Lipo formulations were identical in composition and excipient base, except for the presence of liposomal encapsulation of collagen tripeptides. Consequently, the greater efficacy observed in the Lipo group could reasonably be attributed to the application of liposomal encapsulation, given that no other formulation variables differed between groups. However, as this trial did not include pharmacokinetic or absorption analyses, the precise effects of liposomal encapsulation on peptide stability, biodistribution, and in vivo bioavailability remain to be elucidated. Taken together, these findings suggest that liposomal formulations containing collagen tripeptides represent a promising oral delivery strategy for supporting skin aging‐related parameters. Future investigations should include pharmacokinetic profiling and molecular‐level assessments to further clarify the mechanistic advantages conferred by liposomal delivery.

In conclusion, this randomized, double‐blind, placebo‐controlled clinical study demonstrated that oral supplementation with formulations containing collagen tripeptides was associated with improvements in multiple skin aging–related parameters, including dermal collagen density, hydration, elasticity, wrinkle appearance, and skin tone characteristics. While both collagen formulations produced beneficial effects, the liposomal collagen tripeptide formulation was associated with an earlier onset of improvement and a consistently greater magnitude of change across several objective and subjective endpoints. These findings support the clinical relevance of liposomal delivery as an effective strategy to enhance the functional performance of orally administered formulations containing collagen tripeptides. Collectively, the results suggest that liposomal formulations containing collagen tripeptides may represent a promising oral nutricosmetic strategy for supporting skin structure, appearance, and biomechanical properties in healthy adults.

## Author Contributions

Yung‐Kai Lin contributed to the conception of the study, experimental design, and overall supervision of the research project. Chia‐Hua Liang contributed to study design, coordination of clinical implementation, and data interpretation. Ban‐Chin Huang and Sze‐Huey Lim contributed to formulation design and sample characterization. Yung‐Hsiang Lin contributed to study design, provided conceptual input, and supervised the project. Shu‐Ting Chan and Chi‐Fu Chiang provided experimental guidance, conducted data collection and analysis, and critically revised the manuscript. Yi‐Wen Mao supervised the research project, conducted statistical analyses, and was responsible for drafting and finalizing the manuscript. All authors have read and approved the final version of the manuscript and agree to be accountable for all aspects of the work.

## Funding

This work was supported by TCI Co., Ltd, Taiwan.

## Ethics Statement

This study was approved by the Antai Medical Care Cooperation Antai‐Tian‐Sheng Memorial Hospital Institutional Review Board (Approval No. 24–088‐B) and conducted in accordance with the Declaration of Helsinki. Written informed consent was obtained from all participants prior to enrollment.

## Consent

Written informed consent was obtained from all participants for the use and publication of facial images included in this manuscript. All images were acquired under standardized conditions and anonymized to protect participant identity.

## Conflicts of Interest

The authors declare no conflicts of interest.

## Data Availability

The data supporting the findings of this study are available from the corresponding author upon reasonable request. Due to ethical and privacy considerations, the data are not publicly available.
